# Supplementary material for: Scale-invariant magnetic textures in the strongly correlated oxide NdNiO3
Source: Nat Commun. 2019 Oct 15;10:4568. doi: 10.1038/s41467-019-12502-0 (PMC6794273; doi:10.1038/s41467-019-12502-0)
Supplement: Supplementary file 1 — Supplementary Information [file 41467_2019_12502_MOESM1_ESM.pdf]

Supplementary Information

Scale-invariant magnetic textures in the strongly correlated oxide $NdNiO_3$

Li et al.

Supplementary Information for

Scale-invariant magnetic textures in the strongly correlated oxide $NdNiO_3$

Jiarui Li, Jonathan Pelliciari, Claudio Mazzoli, Sara Catalano, Forrest Simmons, Jerzy T. Sadowski, Abraham Levitan, Marta Gibert, Erica Carlson, Jean-Marc Triscone, Stuart Wilkins, Riccardo Comin

Riccardo Comin
Email: rcomin@mit.edu

Supplementary Information

Supplementary Note 1: Data aquisition

In order to reach the antiferromagnetic (AFM) diffraction peak, at $\mathbf{Q}_{AFM}$ = (¼, ¼, ¼)$_{pc}$, the sample was oriented so that the vertical scattering plane is spanned by the crystallographic vectors [1-10]$_{pc}$ and [111]$_{pc}$. To maximize the scattering intensity, σ polarization was used for all measurements. By raster-scanning the focused X-ray beam across the sample while acquiring the scattering signals, we can record the spatial variations in the magnetic scattering intensity, and consequently map the underlying magnetic texture. The AFM signal was extracted by first subtracting the fluorescence background and then averaging the scattering intensity inside a fixed region of interest (ROI) on the detector. Before acquiring each spatial map, the Cr mask was scanned to ensure all measurements are performed in the same field of view (FOV). The fiducial Cr grid additionally provides a reference frame to correct for the position drift and extract drift-corrected spatial maps over a common spatial range. The position registration enabled by the Cr grid enables to convert the intensity map from the raw pixel grid onto a drift corrected pixel grid by performing an affine transformation followed by point interpolation (Supplementary Fig. 2b).

To recover the signal under the semi-transparent Cr mask, we correct for the photon flux attenuation through the Cr thin layer (Supplementary Fig. 2**c**) [the theoretical transmission factor is 12% for a 143 nm thick Cr, at a photon energy of 852 eV and given a mass density of 7.15 g cm^{-3}]. The data shown in the main text and presented in Fig. 2 have been obtained after removal of the Cr intensity suppression and position drift correction.

The macroscopic beam measurement is performed by moving the sample away from the focus of the zone plates, where the beam footprint is approximately 200 μm in diameter. The temperature was swept with the rate of 1 K min^{-1} while recording the sample temperature and CCD image at a constant rate. The AFM signal was extracted by first subtracting the fluorescence background and then averaging the scattering intensity inside a fixed region of interest (ROI) on the detector. While the spot on the sample is large in this out of focus geometry, the photon beam remains strongly divergent (by virtue of passing through the zone plate focusing element), consequently the width of the diffraction peak on the CCD is largely determined by the divergence of the X-ray beam, with the contribution from the intrinsic spatial correlations being minor. As a result, the AFM correlation lengths could not be directly deconvolved from the diffraction peak linewidth.

Supplementary Note 2: Homogeneous insulating phase well below T_{MIT}

To elucidate whether the AFM inhomogeneity is affected by a nanoscale phase coexistence of metallic and insulating domains[1], we measured the local X-ray absorption spectra (XAS) in the same $NdNiO_3$ film using X-ray PhotoEmission Electron Microscopy (XPEEM) at beamline ESM (21-ID-2) of the National Synchrotron Light Source II. The XAS lineshape at the Ni-L_3 edge shows an ostensible transition across the MIT, over an extended spatial region. When below T_{MIT}, the Ni L_3 XAS exhibits a 2 eV splitting, corresponding to the presence of two inequivalent Ni sites[2]. Figure S3b shows an overlay of multiple representative local XAS spectra from a given field of view. All spectra show prominent insulating feature as opposed to the metallic profile. To obtain a quantified description of the insulating and metallic property, we performed a principal component analysis (PCA) on the XAS map at 100 K, to separate out the metallic and insulating components. The PCA was done by fitting the local XAS spectra, pixel-by-pixel, using a simple linear combination of two model XAS lineshapes representing the metallic ($XAS_{metallic}$, extracted from 300 K absorption spectra) and insulating phase ($XAS_{insulating}$, extracted from absorption spectra at 100 K):

$$XAS(r) = x(r) * XAS_{metallic} + [1 - x(r)] * XAS_{insulating} \quad (1)$$

where the coefficient $x(r)$, constrained within [0,1], represents the local metallicity (Supplementary Fig. 3c). A typical PCA result is shown in the inset of Fig. S3d. We find that >99% of pixels have a metallic character of less than 0.15 [Supplementary Fig. 3d], which reveals a spatially-uniform insulating phase across the FOV when the temperature is well below T_{MIT}. We notice that the stripy textures reported in prior studies[1] were also visible in our measurements and were found to correlate with the atomic terraces at the film surface. This result is consistent with other studies reporting that $NdNiO_3$ films are homogeneously insulating at temperature well below T_{MIT}[3,4]. On the basis of the XPEEM spatial maps, we can rule out a coexistence of the metallic and insulating phases. Consequently, we ascribe the AFM inhomogeneity to the inherent distribution of different magnetic twin domains as described in the main text.

Supplementary Note 3: Memory effect

To quantitatively assess the temperature variations in the domain morphology, we define a local correlation metric to monitor the self-similarity of the probed magnetic textures. The local correlation measure $\rho(\mathbf{r})$ is defined as a conventional cross-correlation of the scattering intensity between two maps, calculated over a region of interest centered at coordinate **r** (see Supplementary Fig. 4a-c). The local spatial filter is a circle of radius of 500 nm, which appears as elliptic because of projection over the sample surface (Fig. 2e). Large and positive values of $\rho(\mathbf{r})$ reflect a higher similarity in the AFM domain morphology across different temperatures (Supplementary Fig. 4d-f). The average cross-correlation measure over the entire field of view ranges around $\bar{\rho} \sim 0.6$, providing direct statistical evidence of a memory effect during thermal cycling. This apparent resilience against temperature variations suggests the occurrence of domain pinning which is robust across the magnetic ordering transition.

Supplementary Note 4: Is it a disorder effect?

To understand the role of disorders in generating spatial inhomogeneity, we simulated a spatial map with 2d uncorrelated percolation model with the same 25% coverage (Supplementary Fig. 5). Domains are only organized within a short range, which is unlike the domain organization observed experimentally and shown in the main text. We also summarized the scaling exponents in Supplementary Table 1, where the same exponents based on 2d uncorrelated percolation model are provided for comparison[5]. Most of the exponents disagree with the uncorrelated percolation model. Therefore, the characteristics of the pattern formation observed in the fractal magnetic texture cannot be attributed solely to the effects of material disorder.

Supplementary Note 5: Signatures of continuously-evolving magnetic order parameter

To visualize the variations in the domain distribution through the AFM transition, we have quantified the magnetic order parameter inside the domains identified in the analysis reported in the maintext. Figure S6 reports three spatial maps (one for each temperature) of the magnetic order parameter inside the AFM domains. These maps reveal a uniform suppression of the magnetic order parameter by a factor ~6 when the temperature is raised from 130 to 180 K, while the underlying domain texture remains largely unaltered. Therefore, our spatially-resolved data support a picture where the magnetic order parameter develops continuously, rather than by nucleation and growth (as could have been inferred from the global temperature evolution of the order parameter, with its hysteretic behavior – see also Fig. 2d in the main text).

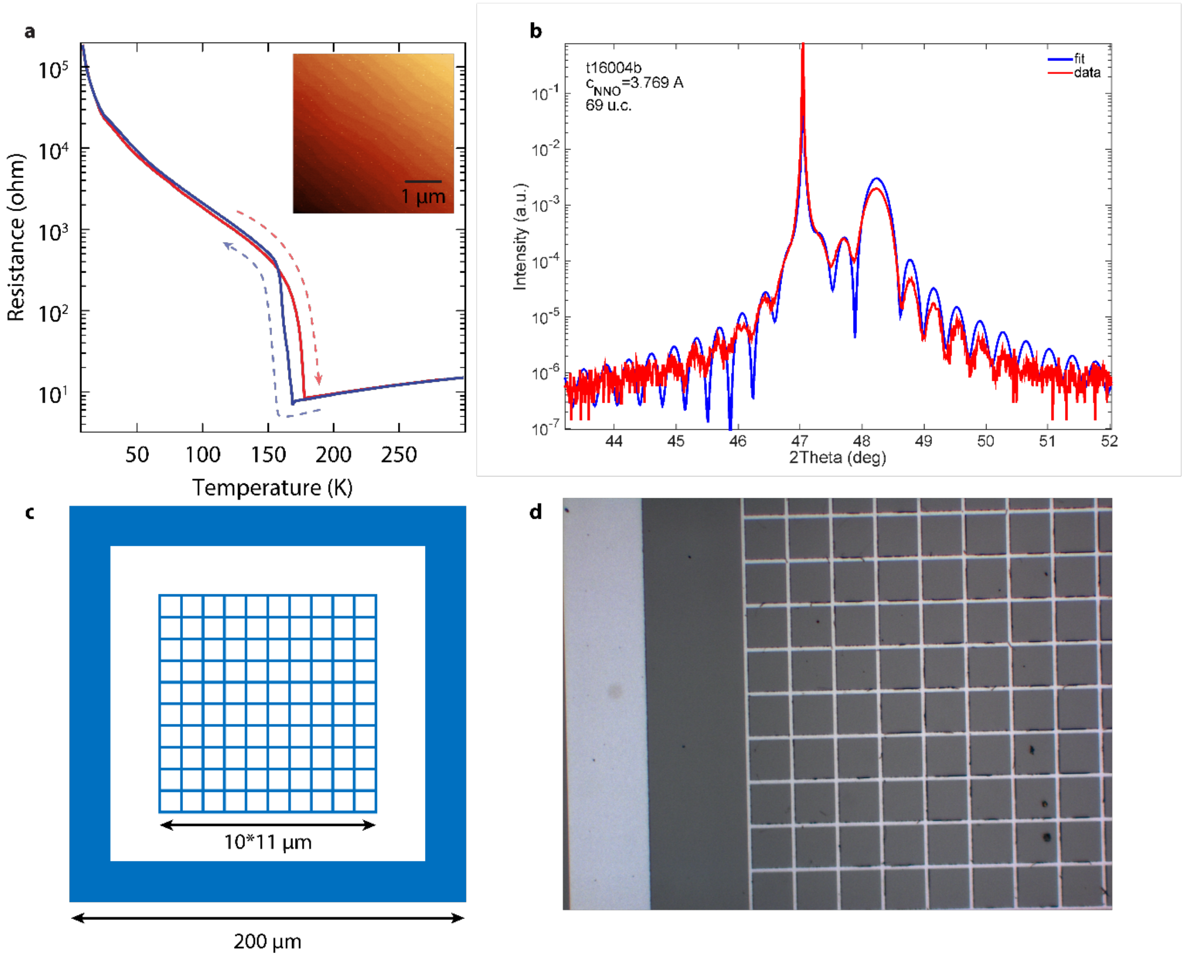

Supplementary Figure 1.

a Resistance versus temperature from the transport measurement. Inset: atomic force microscopy image showing the surface topography. The sample surface is flat and atomic terraces are visible. **b** X-ray reflectivity indicates the film thickness of 26 nm. **c** Schematic drawing of the Cr fiducial mask pattern. The width of the outer frame and the inner grid lines are 20 µm and 1 µm, respectively. The inner grid lines form a 10x10 array of squares with the pitch of 11 µm (1 µm grid line + 10 µm hole). **d** Optical microscopy image of the $NdNiO_3$ film (dark) with overlaid Cr fiducial mask (bright).

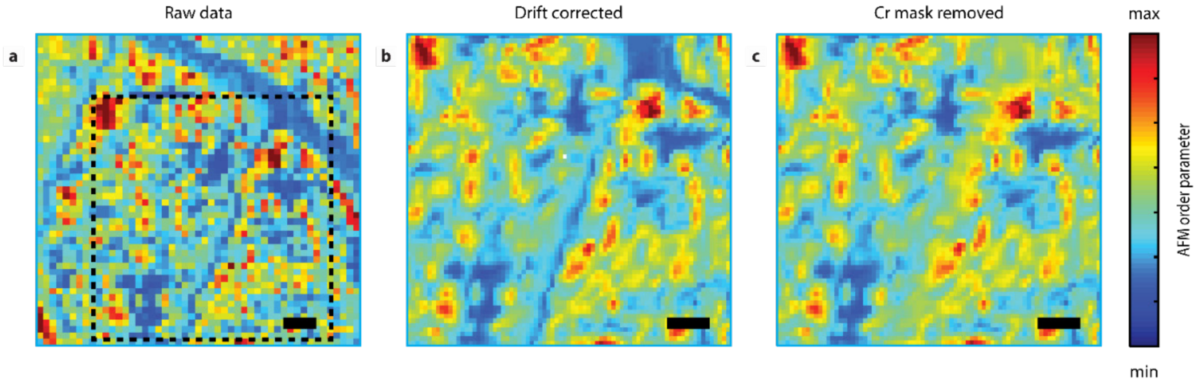

Supplementary Figure 2.

a Acquired nano-RXS images of AFM inhomogeneity at 130 K cooling cycle. The blue lines indicate the intensity contrast created by semi-transparent Cr grid line. Its deviation from a straight line is the result of sample drift. **b** Drift-corrected intensity map from A. The FOV corresponds to the dashed rectangular region in **a**. **c** Cr-recovered AFM domains. The underlying information is corrected for the intensity suppression caused by the incoming and scattered beam attenuation within the Cr overlayer. Scale bar: 1 μm in lateral beam translation coordinates.

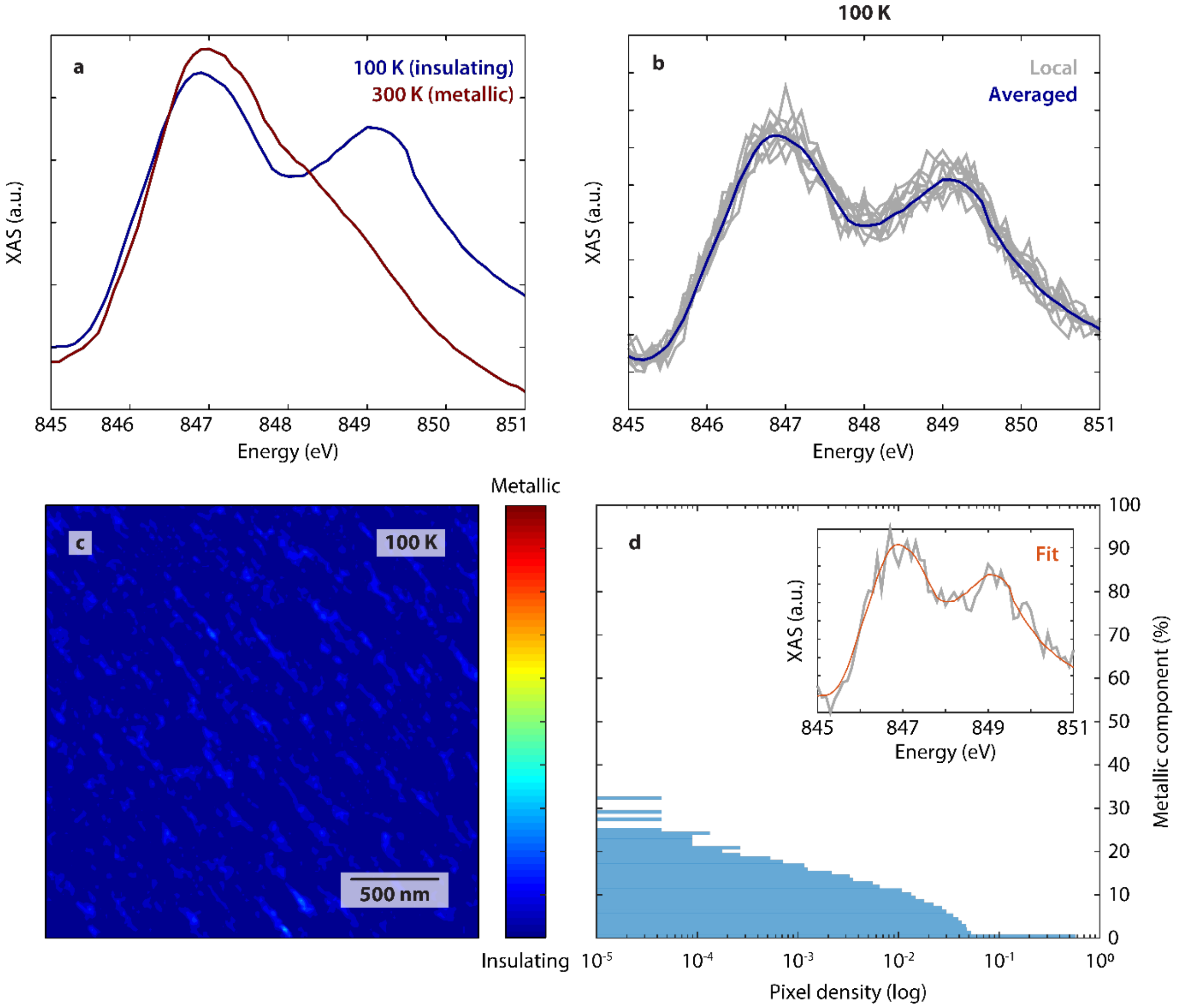

Supplementary Figure 3.

a XAS of NdNiO3 thin film measured at 300 K and 100 K representing metallic and insulating phases. **b** Overlay of multiple local XAS at 100 K. All spectra show very insulating characteristics. **c** Metallic and insulating component map at 100 K, measured by XPEEM. The whole map exhibits strong insulating feature. Stripy textures are results of the atomic terrace on sample surface. Scale bar: 500 nm. **d** Histogram of metallic and insulating component from map in Supplementary Fig. 3**c**. The inset shows a typical PCA result for a single pixel with the metallic component around 15%.

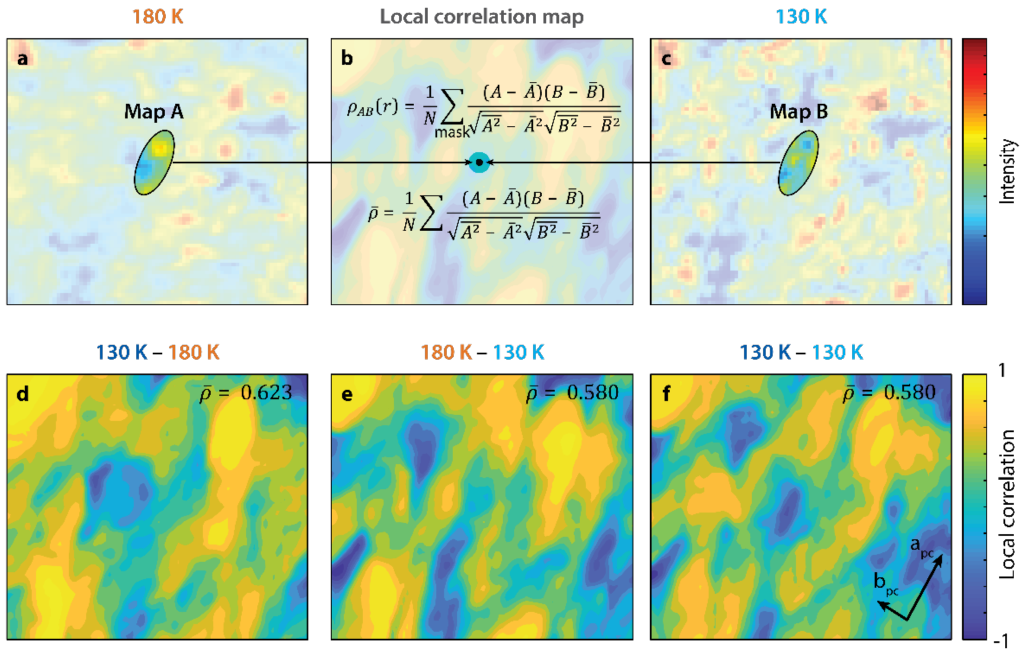

Supplementary Figure 4.

a-c Local correlation map [$\rho(\mathbf{r})$, defined in **b**], calculated as a locally-filtered (with a local circular mask, see **a** and **c**) correlation coefficient between different maps (**a** and **c**). **d-f** Local correlation maps for all temperature pairs. The global cross-correlations $\acute{\rho}$ are all positive and reflect a positively correlated domain distribution against temperature change.

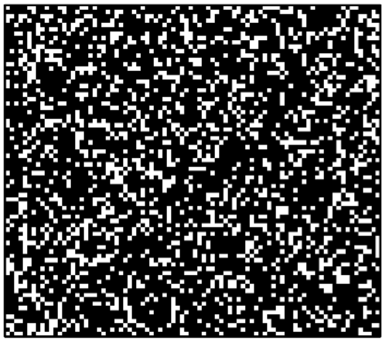

Supplementary Figure 5.
Simulated uncorrelated percolation map at 25% of occupation.

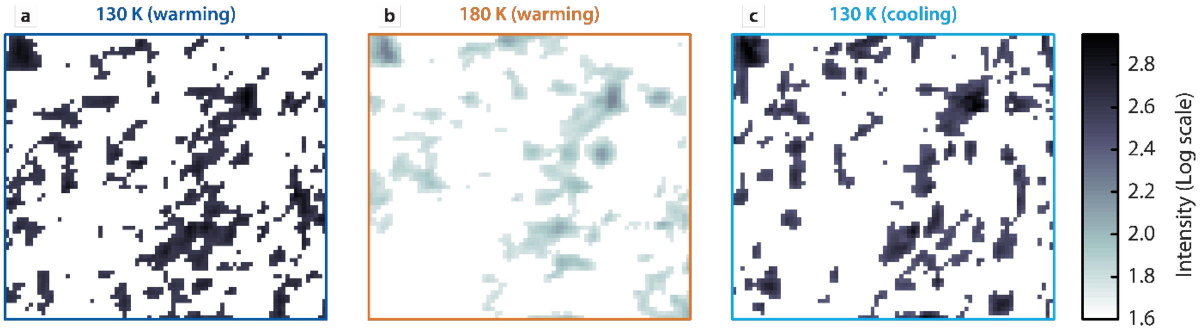

Supplementary Figure 6.

a-c Absolute intensity inside AFM domains (colored region) that we identified in the maintext for three temperatures (log scale). The field of view is the same as Figure 2 (a-c). The spatially-averaged intensity inside AFM domains for three maps are 437, 72, and 347, respectively.

Exponents	τ	d_h	d_v	η
130 K warming	1.21	1.24	1.81	0.178
180 K warming	1.26	1.25	1.83	0.416
130 K cooling	1.29	1.19	1.70	0.376
Average	1.25±0.04	1.23±0.03	1.78±0.07	0.323±0.13
2d- Uncorrelated percolation model	187/91=2.05	7/4=1.75	91/48=1.90	5/24=0.208

Supplementary Table 1.

Fitting results for different maps in Fig. 4. The averaged exponents obtained from a global fit of all data sets are also reported in Fig. 4. Hollow markers represent points excluded from the fit.

References

1. Mattoni, G. *et al.* Striped nanoscale phase separation at the metal-insulator transition of heteroepitaxial nickelates. *Nat. Commun.* **7,** 13141 (2016).
2. Lu, Y. *et al.* Site-Selective Probe of Magnetic Excitations in Rare-Earth Nickelates Using Resonant Inelastic X-ray Scattering. *Phys. Rev. X* **8,** 031014 (2018).
3. Preziosi, D. *et al.* Direct Mapping of Phase Separation across the Metal–Insulator Transition of NdNiO 3. *Nano Lett.* **18,** 2226–2232 (2018).
4. Post, K. W. *et al.* Coexisting first- and second-order electronic phase transitions in a correlated oxide. *Nat. Phys.* **14,** 1056–1061 (2018).
5. Stauffer, D. & Aharony, A. Introduction to Percolation Theory. (1992).
